# Environment, but not genetic divergence, influences geographic variation in colour morph frequencies in a lizard

**DOI:** 10.1186/s12862-015-0442-x

**Published:** 2015-08-08

**Authors:** Claire A. McLean, Devi Stuart-Fox, Adnan Moussalli

**Affiliations:** School of BioSciences, The University of Melbourne, Parkville, VIC 2010 Australia; Sciences Department, Museum Victoria, Carlton Gardens, Melbourne, VIC 3053 Australia

## Abstract

**Background:**

Identifying the causes of intraspecific phenotypic variation is essential for understanding evolutionary processes that maintain diversity and promote speciation. In polymorphic species, the relative frequencies of discrete morphs often vary geographically; yet the drivers of spatial variation in morph frequencies are seldom known. Here, we test the relative importance of gene flow and natural selection to identify the causes of geographic variation in colour morph frequencies in the Australian tawny dragon lizard, *Ctenophorus decresii*.

**Results:**

Populations of *C. decresii* are polymorphic for male throat coloration and all populations surveyed shared the same four morphs but differed in the relative frequencies of morphs. Despite genetic structure among populations, there was no relationship between genetic similarity or geographic proximity and similarity in morph frequencies. However, we detected remarkably strong associations between morph frequencies and two environmental variables (mean annual aridity index and vegetation cover), which together explained approximately 45 % of the total variance in morph frequencies.

**Conclusions:**

Spatial variation in selection appears to play an important role in shaping morph frequency patterns in *C. decresii*. Selection associated with differences in local environmental conditions, combined with relatively low levels of gene flow, is expected to favour population divergence in morph composition, but may be counteracted by negative frequency-dependent selection favouring rare morphs.

**Electronic supplementary material:**

The online version of this article (doi:10.1186/s12862-015-0442-x) contains supplementary material, which is available to authorized users.

## Background

Understanding the processes generating intraspecific phenotypic variation, and how they relate to population divergence and speciation, is a fundamental goal for evolutionary biologists. Polymorphic species, in which multiple, discrete phenotypic variants coexist within a population [1], exhibit extreme, easily measurable, intraspecific phenotypic variation that often varies geographically in the composition and frequency of morphs. Such geographic variation in polymorphism may act as a precursor to speciation if the processes generating geographic variation in morph frequencies also promote phenotypic and genetic differentiation leading to reproductive isolation among populations [2–4]. Indeed, colour polymorphism has been shown to accelerate speciation in birds [3].

One of the most important processes generating morph frequency variation is spatial variation in selection and local adaptation [5]. This has been shown to influence population morph frequencies in multiple species (e.g. [2, 6]), including associations between morph frequencies and broad scale environmental gradients (e.g. [7, 8]). In many cases, however, spatial variation in polymorphism cannot be explained by selection alone [9, 10], and it is therefore likely that genetic drift and gene flow also play a role. Despite a rich history of research on polymorphic species, the relative importance of selection, genetic drift and gene flow for explaining geographic variation in polymorphism remains poorly understood (reviewed in [4]).

Gene flow can be an important determinant of geographic variation in morph frequencies (e.g. [11]) and is expected to constrain local adaptation by introducing individuals from populations experiencing different selective pressures [12, 13]. Therefore, high levels of gene flow may favour the maintenance of polymorphism and homogenise morph frequencies across populations [14]. Conversely, when gene flow is low, geographic variation in polymorphism may reflect local selective pressures [15]. Notably, when local selection is particularly strong, population divergence in morph frequencies can be maintained despite ongoing gene flow [16, 17]. However, local adaptation driven by strong directional selection may be opposed by negative frequency-dependent selection (NFDS), where rare morphs are favoured, generating cyclical changes in morph frequencies over time [18–20]. For example, female preference for novel mates and predator avoidance of rare, unfamiliar colour morphs, likely maintain extreme colour polymorphism in male guppies [21]. Therefore, frequency-dependent selection, spatial variation in selection driven by environmental factors, genetic drift and gene flow can interact in complex ways to influence morph frequencies (e.g. [19]). Consequently, in order to determine the processes generating geographic variation in polymorphism, and their relative contribution, it is necessary to assess the relationship between morph frequencies and multiple factors, including local environmental variables, broad environmental gradients and gene flow.

Although many polymorphic species exhibit population differences in morph composition and frequencies, few studies have examined geographic variation in polymorphism with specific focus on its causes and evolutionary consequences (reviewed in [4]). Here, we evaluate processes that may influence morph frequencies in the Australian tawny dragon lizard, *Ctenophorus decresii*, in which males exhibit striking throat colour variation, both within and among populations [22]. The species comprises two genetically and phenotypically distinct lineages, characterised by unique throat coloration, and corresponding to a polymorphic ‘northern’ lineage and a monomorphic ‘southern’ lineage [22, 23]. There are four discrete throat colour morphs within the ‘northern’ lineage: orange, yellow, orange surrounded by yellow (orange-yellow) and grey (which lacks both orange and yellow; [24]). Data from field mark-recapture studies (M. Yewers and D. Stuart-Fox, unpublished data), and long-term captive breeding experiments [25–27], show that throat coloration is highly heritable, fixed at sexual maturity and does not vary with body size, such that all morphs are observed in the same age/size class [24]. Previous work on *C. decresii* has also detected considerable morph frequency variation among populations [23]; however, this geographic variation is yet to be explicitly documented.

We quantify and compare colour morph frequencies in eight populations of *C. decresii* and test the potential roles of gene flow and selection in shaping morph frequency variation. This system is well suited to studying how the interaction between gene flow and selection influences morph frequencies. Firstly, *C. decresii* is a habitat specialist, restricted to rocky outcrops throughout South Australia, which may form discrete ‘island’ populations with varying degrees of connectivity between populations. Secondly, throat coloration has an important signalling function [27], as males display their throats during both territorial and courtship displays [28], and throat coloration is the main feature distinguishing *C. decresii* from its closest relatives [22]. Additionally, throat coloration in the ‘northern’ and ‘southern’ lineage is locally adapted to different signalling environments. Lineage specific throat colours are more conspicuous to conspecific lizards when viewed against native rock and lichen backgrounds [29]. Finally, colour polymorphism is associated with alternative behavioural strategies. The orange morph exhibits high levels of aggression towards territory intruders in the field, while the grey morph is the least aggressive and the least bold when exposed to a simulated predator attack. The remaining morphs (yellow and orange-yellow males) appear to be intermediates and show aggression that is conditional on the colour morph of the intruder, reacting more aggressively to orange or yellow males than to grey (M. Yewers and D. Stuart-Fox, unpublished data). Therefore, environmentally-driven selection, acting directly on colour or on correlated traits, is expected to influence throat coloration in *C. decresii*. By quantifying the relationship between morph frequencies and genetic and environmental factors, our study provides insights into processes generating and maintaining geographic variation in polymorphism.

## Methods

### Sites and data collection

**Fig. 1:**
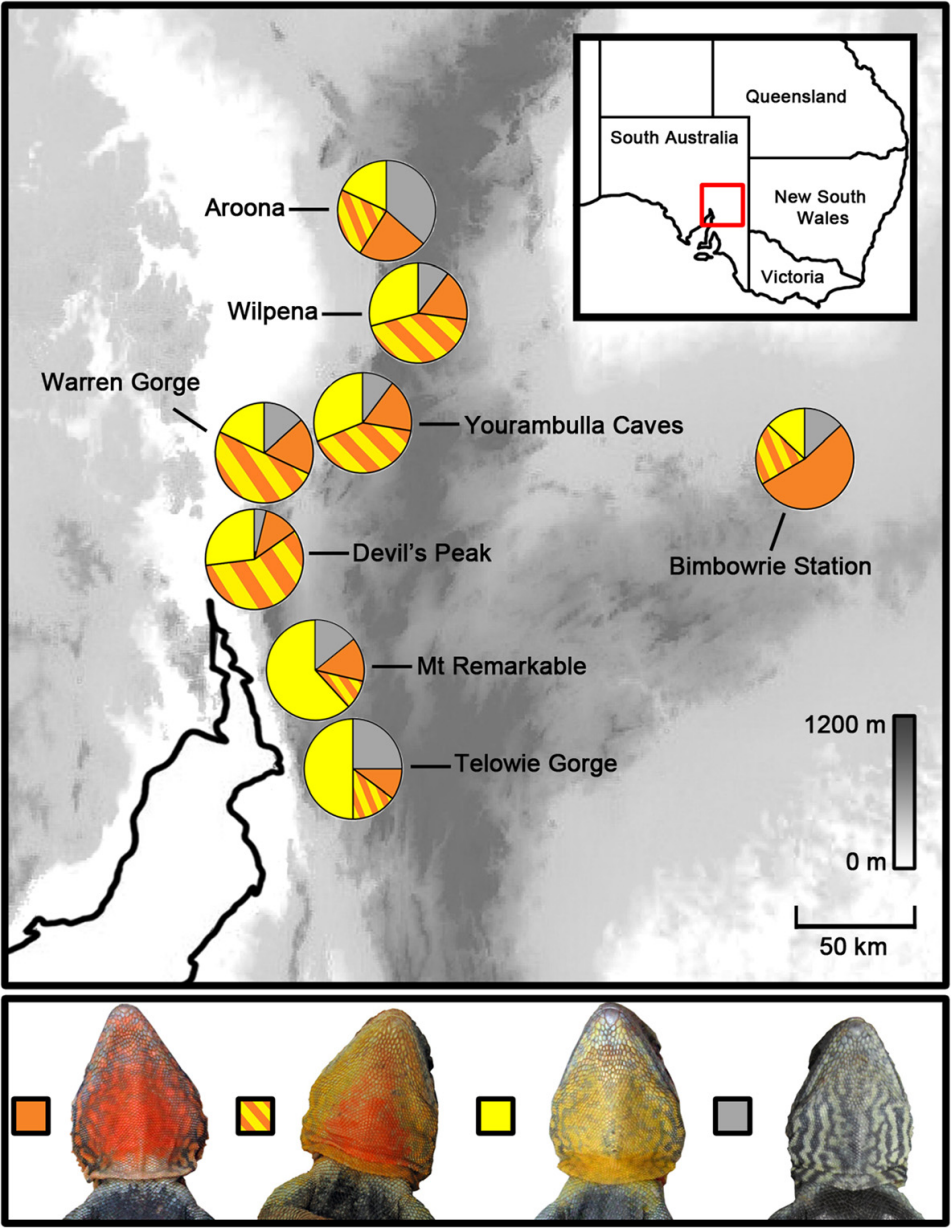
Relative frequencies of orange, orange-yellow, yellow and grey male throat colour morphs in eight populations of *C. decresii*: Aroona (*N* = 22), Wilpena (*N* = 48), Yourambulla Caves (*N* = 29), Warren gorge (*N* = 22), Devil’s Peak (*N* = 26), Bimbowrie Station (*N* = 20), Mt Remarkable (*N* = 21) and Telowie Gorge (*N* = 20). Grey shading represents elevation according to the scale bar

Field work was conducted in South Australia during spring and summer between 2010 and 2012. We sampled eight populations within the polymorphic ‘northern’ lineage of *Ctenophorus decresii* including; Aroona (31°16′39.82" S, 138°36′19.61" E), Wilpena (31°32′24.59" S, 138°36′4.46" E), Yourambulla Caves (31°57′12.99" S, 138°22′24.79" E), Warren Gorge (32°11′15.34" S, 138°00′32.90" E), Devil’s Peak (32°24′57.19" S, 137°59′27.69" E), Mt Remarkable (32°50′39.89" S, 138°03′7.52" E) and Telowie Gorge (33°01′21.43" S, 138°06′24.35" E) in the Flinders Ranges, and Bimbowrie Station (31°58′40.61" S, 140°12′37.65" E) in the Olary Ranges. Within each population, we captured approximately 50 lizards (at least 20 adult males; see Fig. 1 for sample sizes) and took a tissue sample (10 mm tail clip) for subsequent molecular analysis.

Males were photographed using a Canon PowerShot SX1-IS digital camera (saved in RAW format; [30]) and each photograph included a Micnova grey card. Photographs were then linearised for radiance and calibrated (relative to the grey card) to account for differences in illumination between images [30]. A previous study by Teasdale et al. [24] used discriminant function analysis of colour and pattern variables and spectral reflectance data to demonstrate that *C. decresii* males can be reliably classified into discrete throat colour morphs based on the presence or absence of orange and yellow. Using the standardised photographs and these criteria, we visually allocated males into one of four morph types: orange, orange-yellow, yellow or grey. To ensure that morph frequencies did not fluctuate temporally during our study period, two populations (Telowie Gorge and Warren Gorge) were sampled across consecutive years. Morph frequencies were compared among populations and across years using *χ*^2^ tests in R (chisq.test, chisq.post.hoc; [31]).

### Genetic structure and connectivity

Genomic DNA was extracted from tissue with proteinase-K and a GenCatch (TM) Blood and Tissue Genomic Mini-Prep Kit (Epoch Life Sciences, Sugar Land, TX, USA). We amplified eight microsatellite loci previously developed for *C. decresii* (Ctde03, Ctde05, Ctde08, Ctde12, Ctde21 and Ctde45; [23]), or for the congeneric *Ctenophorus pictus* (CP10, CP11; [32, 33]), using published polymerase chain reaction (PCR) protocols [23]. PCR products were sent to Macrogen (Korea) for genotyping and fragment sizes were called using Peak Scanner ver. 1.0 (Applied Biosystems, Foster City, CA, USA). For each locus within each population, we calculated observed and expected heterozygosities and Hardy-Weinberg equilibrium in Arlequin ver. 3.5 (Additional file 1: Table S1 [34]). All loci were checked for the presence of null alleles using Microchecker ver. 2.2.3 [35] and tested for linkage disequilibrium using GenePop ver. 4.2 (Additional file 1: Table S1 [36]). In total we genotyped 30 individuals from each of the eight populations sampled.

Pairwise F_ST_ between populations were calculated in MSA ver. 4.05 [37]. We derived P values based on 10^4^ permutations and applied Bonferonni correction for multiple tests. Isolation by distance was assessed using a simple Mantel test performed in the zt software [38], with pairwise F_ST_ as the independent variable and Euclidian distance between sites as the dependent variable. To investigate genetic structure among populations, we performed a Bayesian clustering analysis in STRUCTURE ver. 2.3.3 [39]. The program uses Markov Chain Monte Carlo (MCMC) sampling to probabilistically assign individuals to populations and determine the most likely number of populations (*K*). We performed 20 independent runs, with random starting seeds, for values of *K* ranging from 1 to 10 with a burn-in length of 1 × 10^5^ iterations and MCMC length of 1 × 10^6^ iterations. We employed the admixture model with correlated allele frequencies and sampling location as a prior. The most likely value of *K* was determined by calculating the rate of change in likelihood between successive *K* values (ΔK; [40]) in Structure Harvester [41]. Given that several hierarchical levels of population structure may exist, and the Δ*K* method detects only the uppermost level [40], we also performed separate STRUCTURE analyses on each of the multi-population clusters inferred in the previous step. This was repeated until the inferred number of clusters (*K*) was one (e.g. [42]). We performed the STRUCTURE analyses with the full microsatellite dataset, and excluding locus CP10 which showed departures from Hardy Weinberg equilibrium across most populations (Additional file 1: Table S1); however, results were qualitatively the same.

### Environmental correlates

To investigate potential environmental correlates of geographic variation in colour morph frequencies, we acquired 0.01 degree (approximately 1 km) resolution climatic and topographic layers from the Atlas of Living Australia (ALA; http://www.ala.org.au; [33]). Given the modest number of populations in our dataset, it was important to minimise the number of variables considered. Therefore, we selected two layers which are likely to be important to *C. decresii* given that the species is a semi-arid, rocky habitat specialist: mean annual aridity index (the monthly ratio of precipitation to potential evaporation and an indicator of dryness) and topographic relief (the range in elevation). Topographic relief provides a measure of the ruggedness of a site, and given that *C. decresii* inhabits rocky outcrops and gorges, this is likely to be a better predictor of habitat quality than elevation alone. Within each 1 km cell, it is calculated as the average change in elevation between adjacent sub-cells of a grid with spacing of approximately 250 m (9 s in latitude and longitude). Using the ALA website, we extracted values for these two variables for each lizard, and calculated the average value within our eight study sites (Additional file 2: Figure S1). Given the resolution of the climatic and topographic layers used, the values obtained were the same for most lizards within a site (lizards within 1 km^2^ of each other); however, the area covered within sites ranged between 0.2–2 km^2^. Furthermore, for each individual captured, the proportion cover of vegetation < 1 m in height and rocks > 50 cm in diameter were visually estimated within a circular quadrat of 3 m radius centred on the point where the lizard was first sighted. These microhabitat variables were assumed to influence territory quality as vegetation structure can affect thermoregulation, predation risk and prey availability (e.g. [43, 44]), and large rocks are used as lookouts and refuges by territorial lizards, including the congeneric ornate dragon, *Ctenophorus ornatus* [45]. With the exception of geographic distance and aridity index (Pearson correlation coefficient = 0.636; PROC CORR, SAS ver. 9.3), there was minimal correlation between the environmental variables examined (Additional file 3: Table S2).

### Determinants of morph frequency variation

We assessed whether geographic variation in morph frequencies in *C. decresii* can be explained by genetic and/or environmental factors using multiple matrix regression as implemented in FSTAT ver. 2.9.3.2 [46]. Although using measures of distance can reduce statistical power, and must be interpreted in relation to the raw variables [47], matrix regressions on distance measures are a powerful method to infer the relative contribution of individual covariates to population differentiation [48]. We included morph frequencies as the response variable and all possible combinations of pairwise F_ST_, geographic distance (to account for spatial autocorrelation), aridity index (aridity), topographic relief (topography), rock cover (rock) and vegetation cover (vegetation) as predictor variables in the models; however, we did not consider models that contained both geographic distance and aridity due to the significant relationship detected between these variables [49]. P values for slope parameters were calculated using 10^4^ permutations.

With the exception of pairwise F_ST_, distance matrices for each of the independent variables were calculated as the Euclidean distance between population pairs [33]. For the dependent variable (morph frequencies) Euclidean distance (between populations *A* and *B*) was calculated as:

dist(*A*, *B*) = √((*A*_O_ – *B*_O_)^2^ + (*A*_OY_ – *B*_OY_)^2^ + (*A*_Y_ – *B*_Y_)^2^ + (*A*_G_ – *B*_G_)^2^),

which incorporates population differences in the frequencies of each of the four colour morphs (O: orange, OY: orange-yellow, Y: yellow, and G: grey). The best fitting models for the data were determined using the corrected Akaike’s information criterion (AICc; [50]). We considered models with AIC_C_ differences (ΔAIC_C_) within five units of the best possible model to be strongly supported. Based on this subset of competing models, we measured the relative importance of each variable by summing the normalised AIC_C_ weights across all models in which a given variable was present (Table 1; [49, 51]). To further aid interpretation of the relationship between morph frequencies and the predictor variables identified in the best model, we performed separate multiple linear regression analyses in R [31] using the raw variables (rather than distances). The frequency of each of the morphs was the dependent variable and the independent variables were the best predictors identified from matrix regressions. Morph frequency data were arcsine transformed and environmental data were log transformed prior to analysis to meet model assumptions.

**Table 1:** Results of multiple regression analyses of distance matrices with divergence in colour morph frequencies (colour) as the dependent variable and showing partial correlation coefficients (*r*_partial_) of each predictor variable included in each model (1–8): pairwise F_ST_, geographic distance (distance), mean annual aridity index (aridity), topographic relief (topography), proportion cover of vegetation <1 m in height (vegetation), and proportion cover of rock >50 cm in diameter (rock). AIC_C_, ΔAIC_C_ and normalised AIC_C_ weights were calculated based on the number of variables in the model (*k*) and the proportion of variance explained by the model (R^2^). Only models with ΔAIC_C_ within five units of the best possible model are shown and the relative importance of variables (RIV) was determined from this subset of models

	F_ST_	distance	aridity	topography	vegetation	rock	*K*	R^2^	AIC_C_	ΔAIC_C_	AIC_C_ weight
RIV	0.106	0.103	0.897	0.172	0.890	0.174					
model											
1			0.640		0.211		3	45.46	146.53	0	0.412
2			0.639		0.211	0.109	4	46.51	148.72	2.19	0.137
3			0.640	−0.099	0.211		4	46.43	148.76	2.23	0.135
4	−0.014		0.640		0.215		4	45.51	149.24	2.71	0.106
5			0.568				2	32.26	150.07	3.55	0.070
6		0.543			0.284		3	37.59	150.30	3.77	0.062
7		0.543					2	29.52	151.19	4.66	0.040
8			0.639	−0.082	0.211	0.109	5	47.18	151.35	4.82	0.037

## Results

### Morph frequency variation

Within Telowie Gorge and Warren Gorge there was no significant difference in morph frequencies across years (Telowie Gorge: *χ*^2^ = 6.98, df = 3, *P* = 0.073; Warren Gorge: *χ*^2^ = 1.62, df = 3, *P* = 0.655), which is not surprising given that longevity is approximately 5 years in the wild (Yewers and Stuart-Fox, unpublished data). Furthermore, the relative rank of morphs was consistent; for example in both years yellow was the most common morph at Telowie Gorge, followed by grey, orange-yellow and then orange. Conversely, at Warren Gorge orange-yellow was the most common morph, followed by yellow, orange and then grey (Fig. 1; Additional file 4: Table S3). Each of the eight populations sampled contained all four colour morphs; however, the frequencies of morphs varied significantly among populations (*χ*^2^ = 243.16, df = 21, *P* < 0.0001; Fig. 1), ranging from 10–53.3 % for orange, 9.5–57.7 % for orange-yellow, 13.3–61.9 % for yellow, and 3.8–36.4 % for grey (Fig. 1). Pairwise comparisons revealed similar morph frequencies in Mt Remarkable and Telowie Gorge (*P* = 0.111) and in Wilpena, Yourambulla Caves, Warren Gorge and Devil’s Peak (P values > 0.05), with the exception of Warren Gorge and Devil’s Peak within this group (*P* = 0.027; Additional file 5: Table S4). Morph frequencies at Mt Remarkable/Telowie Gorge, Aroona and Bimbowrie Station were significantly different from all other populations (all P values < 0.0001; Additional file 5: Table S4).

### Genetic structure and connectivity

**Fig. 2:**
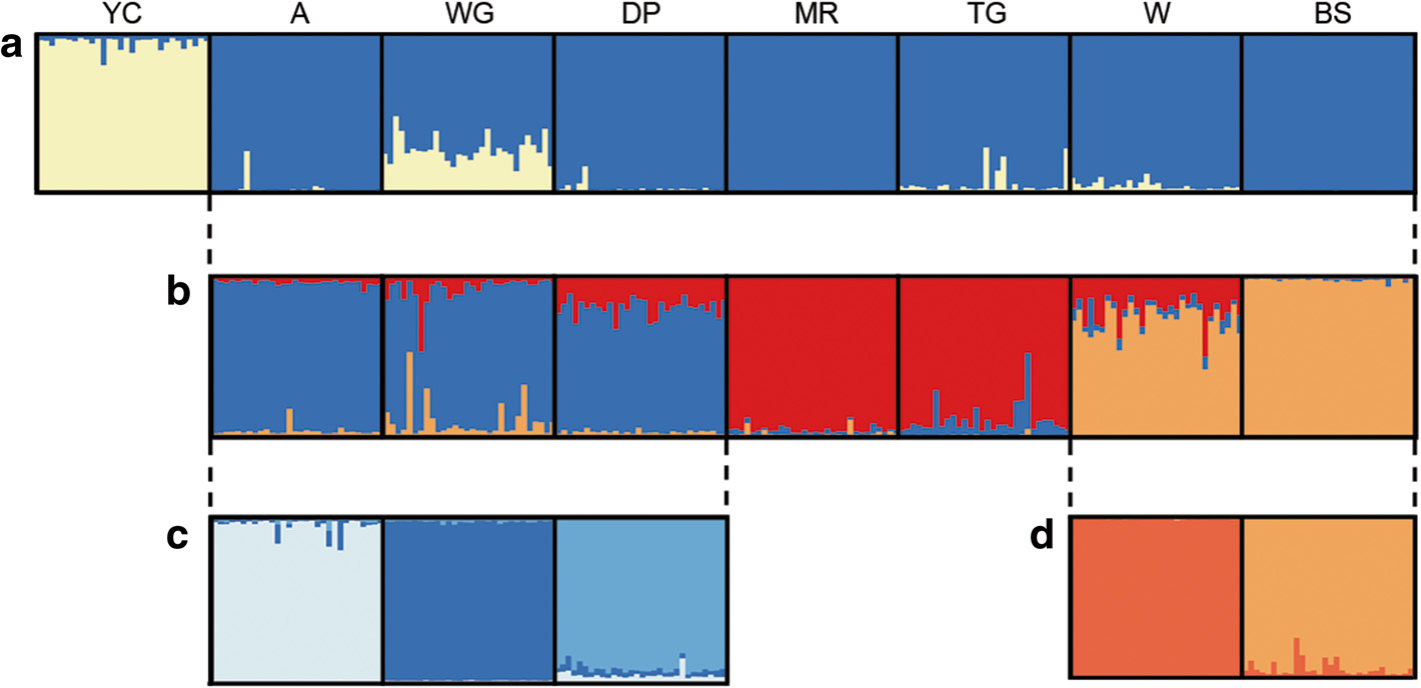
STRUCTURE analysis figures showing hierarchical levels of population structure. Individual assignment probabilities are for **a** *K* = 2 **b** *K* = 3 **c** *K* = 3 and **d** *K* = 2. Populations are: Yourambulla Caves (YC), Aroona (A), Warren Gorge (WG), Devil’s Peak (DP), Mt Remarkable (MR), Telowie Gorge (TG), Wilpena (W), and Bimbowrie Station (BS)

There was genetic subdivision between populations of *C. decresii*. Although pairwise F_ST_ values were low to moderate (ranging from 0.013–0.068), they were statistically significant for all population pairs (with the exception of Mt Remarkable and Telowie Gorge; Additional file 6: Table S5). Genetic similarity did not necessarily reflect geographic proximity as population structure did not conform to a model of isolation by distance (*r*_partial_ = 0.274, *P* = 0.171; Additional file 2: Figure S1). The highest Δ*K* value from the initial STRUCTURE analysis was for *K* = 2 (Δ*K* = 22.01, mean likelihood of *K* = −9901), with Yourambulla Caves forming a separate cluster from all other populations (Fig. 2a). The subsequent analysis excluding Yourambulla Caves inferred three clusters (*K* = 3: Δ*K* = 4.33, mean likelihood of *K* = −8484; Fig. 2b): Aroona/Warren Gorge/Devil’s Peak, Mt Remarkable/Telowie Gorge and Wilpena/Bimbowrie Station. With the exception of Mt Remarkable/Telowie Gorge, these clusters were further separated into individual populations in the final STRUCTURE analyses (Fig. 2c, d).

### Factors affecting morph frequencies

Multiple matrix regression analyses revealed that genetic distance (pairwise F_ST_) was not a significant predictor of divergence in colour morph frequencies in *C. decresii* (*r*_partial_ = 0.026, *P* = 0.470; Additional file 7: Figure S2). Of the other variables included in our model, mean annual aridity index (aridity) and proportion cover of vegetation <1 m in height (vegetation) were the most important predictor variables (RIV = 0.90 and 0.89 respectively) for the observed geographic variation in morph frequencies (aridity: *r*_partial_ = 0.568, *P* = 0.004, vegetation: *r*_partial_ = 0.211, *P* = 0.180; Table 1). The model with the highest AIC_C_ weight combined these two variables (R^2^ = 45.46), and, with the exception of one model (geographic distance; model 7, Table 1), all models with ΔAIC_C_ within five units of the best AICc model included aridity or vegetation or both (Table 1). Despite the moderate correlation between aridity and geographic distance, aridity was a better predictor of morph frequency divergence with RIV = 0.90 compared with RIV = 0.10 for geographic distance (Table 1).

**Fig. 3:**
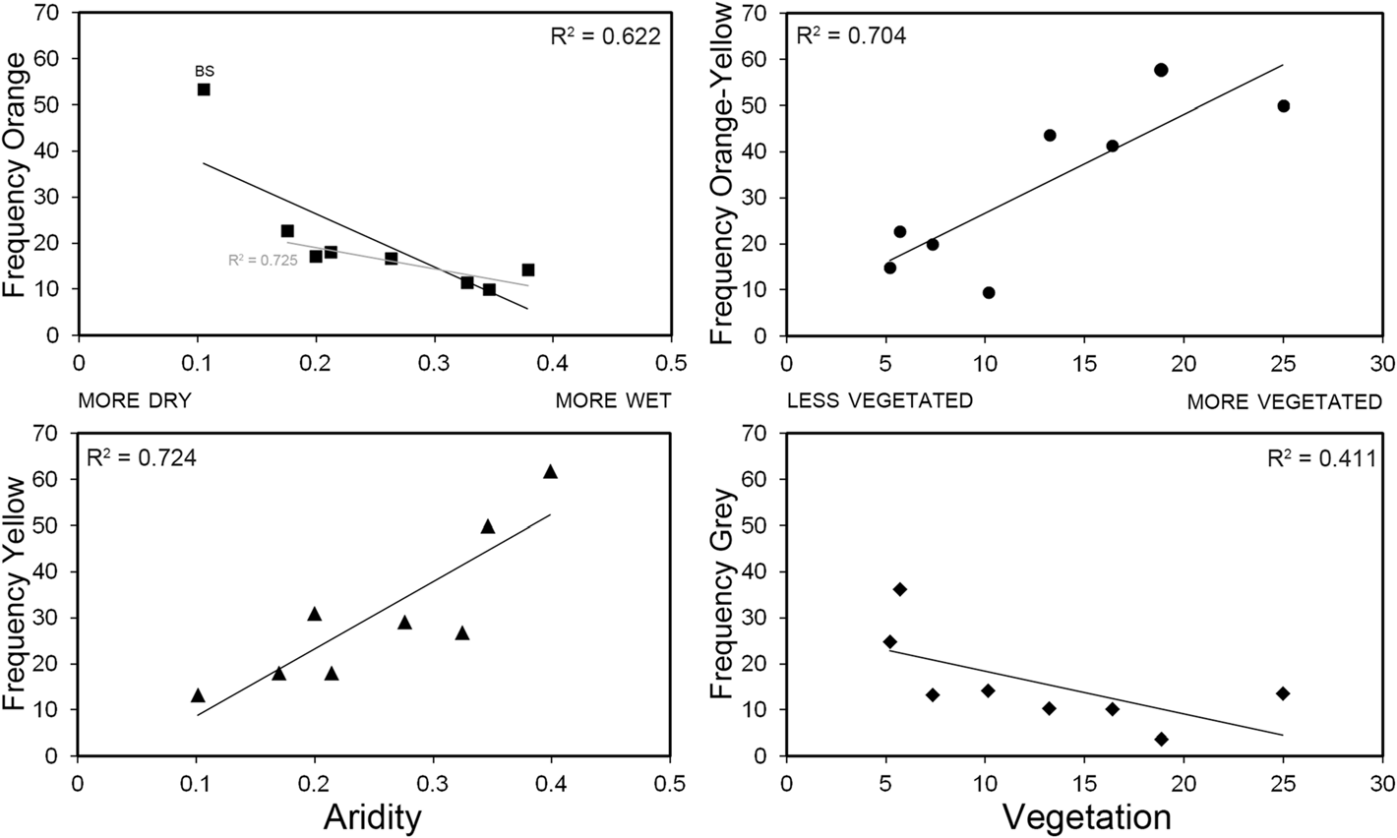
Relationships detected between colour morph frequencies and mean annual aridity index (aridity) and the proportion cover of vegetation <1 m in height (vegetation). Here we plotted the raw variables for which we detected significant relationships in the multiple regression analyses. The analysis for orange morph frequencies was performed with (black) and without (grey) Bimbowrie Station (BS) as this population was identified as an outlier

Linear regressions of the relationship between the frequencies of each morph and the two most informative predictors from the multiple matrix regression models (aridity and vegetation) revealed a consistent pattern of association underpinning the distance-based relationships. The predictor variables explained between 57 and 84 % of the variance in morph frequencies (orange: R^2^ = 0.842, F_2, 5_ = 13.31, *P* = 0.01; orange-yellow: R^2^ = 0.676, F_2, 5_ = 5.22, *P* = 0.06; yellow: R^2^ = 0.756, F_2, 5_ = 7.74, *P* = 0.029; grey: R^2^ = 0.574, F_2, 5_ = 3.36, P = 0.119). Specifically, an increase in aridity was associated with an increase in the proportion of orange individuals (standardised coefficient = −0.892, *P* = 0.004) and a decrease in the proportion of yellow individuals (standardised coefficient = 0.858, *P* = 0.012; Fig. 3; Additional file 8: Table S6). Bimbowrie Station had a particularly high frequency of orange individuals (Grubbs’ outlier test: *G* = 2.32, *P* = 0.002). The relationship between orange morph frequencies and aridity remained significant when Bimbowrie Station was removed from the analysis (standardised coefficient = −0.876, *P* = 0.024). Additionally, there was a trend of higher frequencies of the orange-yellow morph (standardised coefficient = 0.830, *P* = 0.023), and lower frequencies of the grey morph (standardised coefficient = −0.744, *P* = 0.053; Fig. 3; Additional file 8: Table S6), in habitats with a greater proportion cover of vegetation <1 m in height (Fig. 3; Additional file 8: Table S6); however, these relationships were not significant after false discovery rate correction for multiple tests [52].

## Discussion

We quantified colour morph frequencies in eight populations of *C. decresii* and examined their relationship with geographic distance, genetic divergence, broad scale environmental gradients and microhabitat features, to elucidate the causes of geographic variation in polymorphism. Our analyses revealed genetic structure among populations, but no relationship between geographic proximity or genetic similarity and similarity in morph frequencies. However, we found a strong association between divergence in morph frequencies and divergence in environmental variables (aridity index and vegetation cover <1 m in height), with the proportion of variance explained being remarkably high for the relatively simple models and modest number of populations considered here (accounting for approximately 45 % of the variance in morph frequencies). This indicates a potentially important role for environmentally driven selection in shaping morph frequencies in *C.* decresii, as is also observed in multiple other species, including land snails, *Cepaea nemoralis* [53], damselflies, *Ischnura senegalans* [54], rock pocket mice, *Chaetodipus intermedius* [6] and owls [8]. Conversely, environmentally driven selection has been shown to be an unlikely explanation for geographic variation in morph frequencies in several polymorphic lizard species (*Podarcis gaigae* [11], *Anolis sagrei* [9], *Urosaurus ornatus* [55]).

Increasing aridity was associated with a higher proportion of orange males, and fewer yellow males. Broad environmental gradients such as aridity could influence morph frequencies directly by affecting the relative fitness of morphs (e.g. [6, 56]) and/or indirectly by altering intra- and inter-specific interactions (e.g. [57, 58]). Given that discrete colour morphs are often genetically correlated with other traits such as behaviour, physiology and life-history, selection may act on these correlated traits which make certain morphs better adapted to different environments. In *C. decresii*, colour morphs appear to differ in behavioural strategies. In particular, a study performed at Yourambulla Caves found that orange males react more aggressively to model intruders (M. Yewers and D. Stuart-Fox, unpublished data). Therefore, the orange morph may adopt a dominant behavioural strategy, as is also observed in other polymorphic lizard species (*Uta stansburiana*, [20]; *Podarcis melisellensis*, [59]; *Sceloporus consobrinus*, [60]), and being more aggressive may provide a competitive advantage in more arid habitats where resources are scarce. Additionally, morphs may also differ in other physiological and/or life history traits that are yet to be quantified. For example, colour morphs of the common wall lizard, *Podarcis muralis*, differ in their immune response to parasites [61] and sensitivity to stress [62]. Further work is needed to determine the full suite of correlated traits in *C. decresii* and the complex interaction between morphs and their environment.

We also detected a trend of fewer grey males, and more orange-yellow males, in populations with a greater proportion cover of vegetation. The morphs do not differ in microhabitat preferences, including vegetation cover [24]; however, vegetation cover may affect signal efficacy by altering light conditions [63] or obscuring colour signals. For example, in the lizard *Anolis cristatellus*, dewlap colour is adapted to habitat type, and populations from more mesic habitats have brighter dewlaps [64]. In *C. decresii*, the grey morph is the least conspicuous, whereas the orange and orange-yellow morphs are the most conspicuous against natural rock backgrounds to the visual system of a conspecific lizard [24, 29]. Furthermore, orange and yellow coloration should maximize conspicuousness in small patches of sunlight where the light environment is rich in long wavelengths [63]. Consequently, orange-yellow throat coloration may be a more effective colour signal than grey throat coloration in highly vegetated habitats, as it is more conspicuous to females and rival males. Vegetation cover could also alter the abundance and species composition of predators, which may interact with selection for signal efficacy to influence the relative fitness of colour morphs [65, 66]. Given the strong relationship between morph frequencies and both aridity and vegetation cover identified here, it would be illuminating to document associated geographic variation in predator species and the relative conspicuousness of morphs in different habitats.

In contrast to the strong relationship between morph frequencies and environmental factors, there was no relationship with genetic divergence or geographic distance. Differences in morph frequencies between geographically adjacent or closely related populations may arise when neighbouring populations differ strongly in their environment, and local conditions select against immigrants (a process termed ‘Isolation By Adaptation’, and a critical component of ecological speciation [15, 67, 68]). Notably, neighbouring populations of *C. decresii* differed markedly in aridity (e.g. Aroona and Wilpena), vegetation cover (e.g. Mt Remarkable and Devil’s Peak), or both (e.g. Warren Gorge and Devil’s Peak). Furthermore, local directional selection favouring common morphs may be countered by NFDS favouring rare morphs [18–20]. NFDS could also potentially reduce or magnify apparent gene flow by favouring or selecting against immigrants. Specifically, NFDS will favour immigrants belonging to locally rare morphs and select against locally common morphs, which are expected to constitute the largest proportion of migrants between populations with similar morph ranks. For example, Wilpena and Yourambulla Caves have almost identical morph frequencies and are geographically proximate with no obvious barriers to gene flow; however, these populations are genetically distinct. The potential effect of NFDS on morph-specific migration will ultimately depend on the magnitude of differences in morph frequencies between populations and the strength of NFDS in relation to local directional selection. Lastly, morph frequencies in neighbouring populations may be affected by phenotypic or behavioural differences between morphs (e.g. [69]). For instance, morphs may differ in dispersal, such as in the colour polymorphic side-blotched lizard, *Uta stansburiana* [70]. Therefore, future research on the *C. decresii* system should focus on the role of NFDS in maintaining colour polymorphism and whether morph-specific behavioural strategies are associated with dispersal patterns.

Assessing how gene flow and selection interact to shape morph frequencies within a species has broad implications for understanding whether or not polymorphism is likely to promote speciation (reviewed in [4]). Spatial variation in ecological selection coupled with low or absent gene flow between populations (or selection against migrants) is expected to facilitate population divergence through the eventual fixation of different morphs in different populations [15]. In *C. decresii*, despite conditions potentially favouring divergence associated with local environmental conditions, populations did not differ in morph composition (i.e. the number and type of morphs). Rare morphs may be maintained by migration between populations; however, gene flow between populations varied, with some populations (e.g. Yourambulla Caves) appearing to have low or absent gene flow. Hence, an alternative explanation is that morphs are under NFDS, a powerful evolutionary force maintaining polymorphism (by favouring rare morphs, including in lizards e.g. *Uta stansburiana*, [20]; *Anolis sagrei*, [9]), thus countering the influence that local environmental conditions may have on morph frequencies. Assessing morph frequency changes over time would provide insight into whether NFDS also maintains polymorphism in *C. decresii*.

## Conclusions

Morph frequency patterns in *C. decresii* did not reflect genetic divergence or geographic distance. By contrast, we detected strong relationships between colour morph frequencies and a broad environmental gradient (mean annual aridity index) and microhabitat feature (vegetation cover). Furthermore, while morph frequencies differed significantly across populations, all populations surveyed contained the same four morph types. Selection associated with geographic variation in environmental factors is expected to favour local adaptation and population divergence in morph composition, but may be counteracted by NFDS. Ultimately, it is the interplay between NFDS and divergent natural selection that is likely to be the most important determinant of the potential for speciation in geographically variable polymorphic species.

## Data Accessibility

The data set supporting the results of this article is available in the LabArchives repository, http://dx.doi.org/10.6070/H42805MZ.

## Additional files

Additional file 1: Table S1. Characteristics of eight microsatellite loci, screened across thirty individuals from eight populations of *Ctenophorus decresii*. Significant linkage disequilibrium was detected between Ctde08 and Ctde45 across all populations. Statistically significant values are bold and italicised, * indicates possible null alleles. (PDF 197 kb) Additional file 2: Figure S1. Mean annual aridity index and topographic relief across the range of northern *C. decresii* with values for Aroona (A), Bimbowrie Station (BS), Devil’s Peak (DP), Mt Remarkable (MR), Telowie Gorge (TG), Wilpena (W), Warren Gorge (WG) and Yourambulla Caves (YC). (PDF 311 kb) Additional file 3: Table S2. Correlations among geographic distance (distance), mean annual aridity index (aridity), topographic relief (topography), proportion cover of vegetation <1 m in height (vegetation), and proportion cover of rocks >50 cm in diameter. Pearson correlation coefficients are below the diagonal and corresponding P values are above the diagonal. Statistically significant relationships after false discovery rate correction for multiple tests are bold and italicised [52]. (PDF 85 kb) Additional file 4: Table S3. Relative frequencies of male colour morphs at Telowie Gorge and Warren Gorge in 2010 and 2011. (PDF 84 kb) Additional file 5: Table S4. Test for significant differences in male morph frequencies among all pairs of populations in a chi-square test (A: Aroona, W: Wilpena, YC: Yourambulla Caves, WG: Warren Gorge, DP: Devil’s Peak, BS: Bimbowrie Station, MR: Mt Remarkable, TG: Telowie Gorge). Statistically significant values after false discovery rate correction for multiple tests are bold and italicised [52]. (PDF 146 kb) Additional file 6: Table S5. Pairwise corrected F_ST_ between Aroona (A), Wilpena (W), Yourambulla Caves (YC), Warren Gorge (WG), Devil’s Peak (DP), Bimbowrie Station (BS), Mt Remarkable (MR) and Telowie Gorge (TG) calculated from eight microsatellite loci (below the diagonal) and P values after sequential Bonferonni correction for multiple tests (above the diagonal). (PDF 139 kb) Additional file 7: Figure S2. Figures showing weak relationships between pairwise F_ST_ and geographic distance (a), and pairwise F_ST_ and divergence in colour morph composition (b). (PDF 152 kb) Additional file 8: Table S6. Results of four separate multiple regression analyses with the frequency of each colour morph (orange, orange-yellow, yellow and grey) as the dependent variables and mean annual aridity index (aridity) and proportion cover of vegetation <1 m in height (vegetation) as the predictor variables. Statistically significant relationships after false discovery rate correction for multiple tests are bold and italicised [52]. (PDF 88 kb)

## Abbreviations

AIC: Akaike’s information criterion
ALA: Atlas of living Australia
NFDS: Negative frequency-dependent selection
RIV: Relative importance of variables
