# Supplementary material for: Environment, but not genetic divergence, influences geographic variation in colour morph frequencies in a lizard
Source: BMC Evol Biol. 2015 Aug 8;15:156. doi: 10.1186/s12862-015-0442-x (PMC4528382; doi:10.1186/s12862-015-0442-x)
Supplement: Additional file 1: Table S1. — Characteristics of eight microsatellite loci, screened across thirty individuals from eight populations of Ctenophorus decresii. Significant linkage disequilibrium was detected between Ctde08 and Ctde45 across all populations. Statistically significant values are bold and italicised, * indicates possible null alleles. (PDF 197 kb) [file 12862_2015_442_MOESM1_ESM.pdf]

Table S1. Characteristics of 8 microsatellite loci, screened across 30 individuals from 8 populations of *Ctenophorus decresii*. Significant linkage disequilibrium was detected between Ctde08 and Ctde45 across all populations. Statistically significant values are bold and italicised, * indicates possible null alleles.

Population	Locus	Size Range	N_A	H_O	H_E	HWE
Aroona	Ctde03	283–363	18	0.867	0.913	0.692
	Ctde05	183–241	11	0.833	0.852	0.486
	Ctde08	148–240	11	0.867	0.891	0.386
	Ctde12	227–309	19	0.900	0.937	0.755
	Ctde21	291–349	13	0.867	0.907	0.229
	Ctde45	149–212	8	0.767	0.841	0.115
	CP10	105–248	16	0.633	0.911	***0.000****
	CP11	136–148	4	0.500	0.585	0.783
Wilpena	Ctde03	273–371	19	0.833	0.934	0.105
	Ctde05	167–243	15	0.867	0.919	0.406
	Ctde08	152–214	13	0.700	0.858	0.159*
	Ctde12	223–305	19	0.933	0.942	0.868
	Ctde21	287–393	18	0.833	0.884	0.266
	Ctde45	153–256	12	0.700	0.855	0.142*
	CP10	113–186	22	0.700	0.937	***0.000****
	CP11	137–154	7	0.500	0.485	0.140
Yourambulla Caves	Ctde03	261–355	12	0.700	0.850	0.321*
	Ctde05	167–261	14	0.933	0.910	0.786
	Ctde08	144–214	15	0.933	0.929	0.348

	Ctde12	239–313	16	0.833	0.915	0.477
	Ctde21	287–369	10	0.633	0.824	***0.014****
	Ctde45	145–212	11	0.793	0.898	0.089
	CP10	120–148	14	0.633	0.885	***0.000****
	CP11	142–150	4	0.300	0.518	***0.007****
Devil's Peak	Ctde03	291–399	16	0.933	0.929	0.128
	Ctde05	167–245	13	0.900	0.887	0.618
	Ctde08	148–210	9	0.667	0.855	0.193*
	Ctde12	227–397	18	0.900	0.937	0.057
	Ctde21	287–373	12	0.633	0.739	***0.041***
	Ctde45	149–208	8	0.600	0.794	***0.043****
	CP10	113–188	25	0.724	0.948	***0.003****
	CP11	138–150	5	0.667	0.603	0.753
Warren Gorge	Ctde03	249–363	19	0.933	0.938	0.092
	Ctde05	167–237	14	0.867	0.925	0.455
	Ctde08	152–200	11	0.867	0.878	0.757
	Ctde12	247–317	14	0.833	0.918	0.279
	Ctde21	287–373	14	0.800	0.859	***0.011***
	Ctde45	153–208	10	0.900	0.841	0.392
	CP10	118–172	18	0.667	0.935	***0.000****
	CP11	140–152	6	0.667	0.600	0.600
Bimbowrie Station	Ctde03	257–355	15	0.933	0.929	0.751
	Ctde05	167–231	15	0.867	0.914	0.144
	Ctde08	148–204	12	0.867	0.889	0.076

	Ctde12	227–313	14	0.767	0.920	***0.006****
	Ctde21	291–369	11	0.800	0.791	0.739
	Ctde45	149–204	10	0.833	0.855	0.099
	CP10	126–180	17	0.483	0.912	***0.000****
	CP11	138–148	5	0.400	0.402	0.515
Mt Remarkable	Ctde03	261–359	19	0.900	0.942	0.230
	Ctde05	167–265	16	0.867	0.903	0.730
	Ctde08	152–200	9	0.833	0.838	***0.013***
	Ctde12	227–321	20	1.000	0.944	0.606
	Ctde21	287–361	13	0.867	0.908	0.146
	Ctde45	153–200	8	0.767	0.777	0.487
	CP10	113–180	17	0.700	0.936	***0.001****
	CP11	138–150	6	0.600	0.494	0.967
Telowie Gorge	Ctde03	271–367	18	0.900	0.933	0.428
	Ctde05	167–255	17	0.900	0.927	0.201
	Ctde08	144–206	8	0.700	0.826	0.082
	Ctde12	227–317	19	0.867	0.936	0.331
	Ctde21	287–357	10	0.821	0.819	0.631
	Ctde45	145–204	7	0.600	0.741	0.220
	CP10	113–190	22	0.800	0.941	0.220*
	CP11	138–150	5	0.533	0.567	0.362
