# Supplementary material for: Environment, but not genetic divergence, influences geographic variation in colour morph frequencies in a lizard
Source: BMC Evol Biol. 2015 Aug 8;15:156. doi: 10.1186/s12862-015-0442-x (PMC4528382; doi:10.1186/s12862-015-0442-x)

Fig. S1. Mean annual aridity index and topographic relief across the range of northern *C. decresii* with values for Aroona (A), Bimbowrie Station (BS), Devil's Peak (DP), Mt Remarkable (MR), Telowie Gorge (TG), Wilpena (W), Warren Gorge (WG) and Yourambulla Caves (YC).

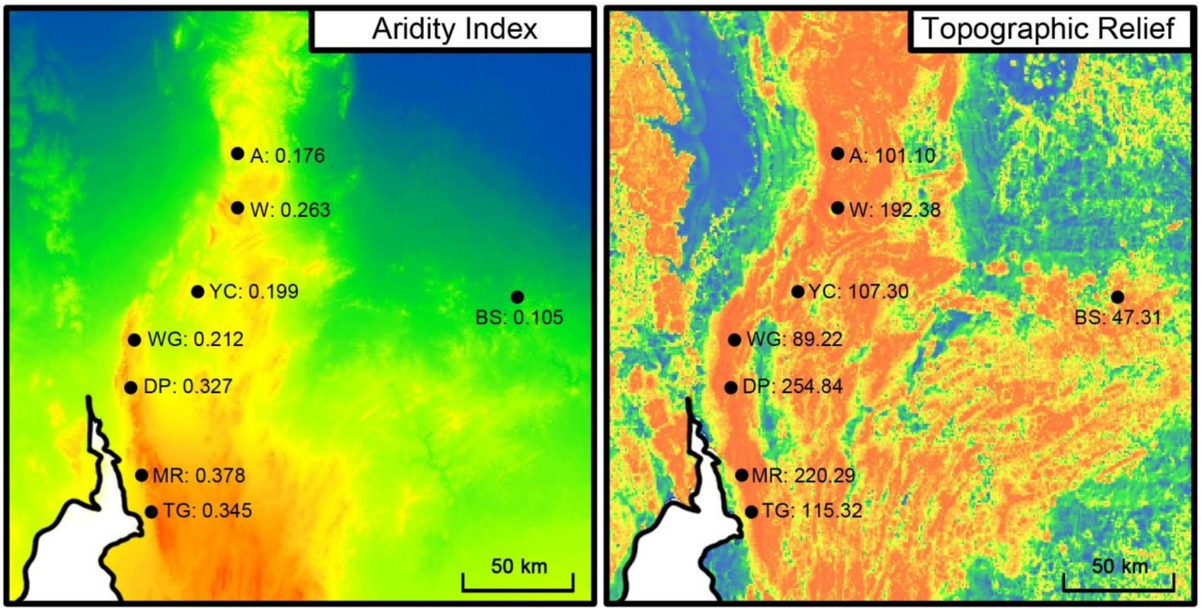

Supplement: Additional file 2: Figure S1. — Mean annual aridity index and topographic relief across the range of northern C. decresii with values for Aroona (A), Bimbowrie Station (BS), Devil’s Peak (DP), Mt Remarkable (MR), Telowie Gorge (TG), Wilpena (W), Warren Gorge (WG) and Yourambulla Caves (YC). (PDF 311 kb) [file 12862_2015_442_MOESM2_ESM.pdf]
