# Supplementary material for: Environment, but not genetic divergence, influences geographic variation in colour morph frequencies in a lizard
Source: BMC Evol Biol. 2015 Aug 8;15:156. doi: 10.1186/s12862-015-0442-x (PMC4528382; doi:10.1186/s12862-015-0442-x)
Supplement: Additional file 3: Table S2. — Correlations among geographic distance (distance), mean annual aridity index (aridity), topographic relief (topography), proportion cover of vegetation <1 m in height (vegetation), and proportion cover of rocks >50 cm in diameter. Pearson correlation coefficients are below the diagonal and corresponding P values are above the diagonal. Statistically significant relationships after false discovery rate correction for multiple tests are bold and italicised [52]. (PDF 85 kb) [file 12862_2015_442_MOESM3_ESM.pdf]

Table S2. Correlations among geographic distance (distance), mean annual aridity index (aridity), topographic relief (topography), proportion cover of vegetation <1m in height (vegetation), and proportion cover of rocks >50cm in diameter. Pearson correlation coefficients are below the diagonal and corresponding P values are above the diagonal. Statistically significant relationships after false discovery rate correction for multiple tests are bold and italicised (Verhoeven et al., 2005).

	distance	aridity	topography	vegetation	rock
distance	-	***0.0003***	0.7121	0.5105	0.1889
aridity	0.6359	-	0.0169	0.2037	0.9246
topography	0.0730	0.4477	-	0.4783	0.4634
vegetation	-0.1297	-0.2477	-0.1397	-	0.8706
rock	0.2558	0.0187	-0.1444	0.0322	-
