# Supplementary material for: Environment, but not genetic divergence, influences geographic variation in colour morph frequencies in a lizard
Source: BMC Evol Biol. 2015 Aug 8;15:156. doi: 10.1186/s12862-015-0442-x (PMC4528382; doi:10.1186/s12862-015-0442-x)
Supplement: Additional file 4: Table S3. — Relative frequencies of male colour morphs at Telowie Gorge and Warren Gorge in 2010 and 2011. (PDF 84 kb) [file 12862_2015_442_MOESM4_ESM.pdf]

Table S4. Relative frequencies of male colour morphs at Telowie Gorge and Warren Gorge in 2010 and 2011.

Colour Morph	Telowie Gorge 2010 (N = 15)	Telowie Gorge 2011 (N = 20)	Warren Gorge 2010 (N = 22)	Warren Gorge 2011 (N = 48)
Orange	5.88	10	18.18	16.67
Orange-Yellow	5.88	15	50	47.92
Yellow	64.71	50	18.18	25
Grey	23.53	25	13.64	10.42
