# Supplementary material for: Environment, but not genetic divergence, influences geographic variation in colour morph frequencies in a lizard
Source: BMC Evol Biol. 2015 Aug 8;15:156. doi: 10.1186/s12862-015-0442-x (PMC4528382; doi:10.1186/s12862-015-0442-x)
Supplement: Additional file 5: Table S4. — Test for significant differences in male morph frequencies among all pairs of populations in a chi-square test (A: Aroona, W: Wilpena, YC: Yourambulla Caves, WG: Warren Gorge, DP: Devil’s Peak, BS: Bimbowrie Station, MR: Mt Remarkable, TG: Telowie Gorge). Statistically significant values after false discovery rate correction for multiple tests are bold and italicised [52]. (PDF 146 kb) [file 12862_2015_442_MOESM5_ESM.pdf]

Table S3. Test for significant differences in male morph frequencies among all pairs of populations in a chi-square test (A: Aroona, W: Wilpena, YC: Yourambulla Caves, WG: Warren Gorge, DP: Devil's Peak, BS: Bimbowrie Station, MR: Mt Remarkable, TG: Telowie Gorge). Statistically significant values after false discovery rate correction for multiple tests are bold and italicised (Verhoeven et al., 2005).

	A	W	YC	WG	DP	BS	MR	TG
A	-							
W	< ***0.0001***	-						
YC	< ***0.0001***	0.9891	-					
WG	***0.0001***	0.3025	0.1718	-				
DP	< ***0.0001***	0.1502	0.0874	***0.0273***	-			
BS	***< 0.0001***	***< 0.0001***	***< 0.0001***	***< 0.0001***	***< 0.0001***	-		
MR	***<0.0001***	***< 0.0001***	***< 0.0001***	***< 0.0001***	***< 0.0001***	***< 0.0001***	-	
TG	***< 0.0001***	***< 0.0001***	***< 0.0001***	***< 0.0001***	***< 0.0001***	***< 0.0001***	0.1108	-
