# Supplementary material for: Environment, but not genetic divergence, influences geographic variation in colour morph frequencies in a lizard
Source: BMC Evol Biol. 2015 Aug 8;15:156. doi: 10.1186/s12862-015-0442-x (PMC4528382; doi:10.1186/s12862-015-0442-x)
Supplement: Additional file 6: Table S5. — Pairwise corrected FST between Aroona (A), Wilpena (W), Yourambulla Caves (YC), Warren Gorge (WG), Devil’s Peak (DP), Bimbowrie Station (BS), Mt Remarkable (MR) and Telowie Gorge (TG) calculated from eight microsatellite loci (below the diagonal) and P values after sequential Bonferonni correction for multiple tests (above the diagonal). (PDF 139 kb) [file 12862_2015_442_MOESM6_ESM.pdf]

Table S5. Pairwise corrected F_{ST} between Aroona (A), Wilpena (W), Yourambulla Caves (YC), Warren Gorge (WG), Devil's Peak (DP), Bimbowrie Station (BS), Mt Remarkable (MR) and Telowie Gorge (TG) calculated from eight microsatellite loci (below the diagonal) and P values after sequential Bonferonni correction for multiple tests (above the diagonal).

	A	W	YC	WG	DP	BS	MR	TG
A	-	0.0028	0.0028	0.0028	0.0028	0.0028	0.0028	0.0028
W	0.039	-	0.0028	0.0028	0.0028	0.0028	0.0028	0.0028
YC	0.064	0.044	-	0.0028	0.0028	0.0028	0.0028	0.0028
WG	0.035	0.030	0.034	-	0.0028	0.0028	0.0028	0.0028
DP	0.029	0.031	0.062	0.022	-	0.0028	0.0028	0.0028
BS	0.045	0.034	0.068	0.044	0.043	-	0.0028	0.0028
MR	0.041	0.035	0.055	0.028	0.027	0.043	-	ns
TG	0.035	0.034	0.057	0.027	0.026	0.035	0.013	-
