# Supplementary material for: Environment, but not genetic divergence, influences geographic variation in colour morph frequencies in a lizard
Source: BMC Evol Biol. 2015 Aug 8;15:156. doi: 10.1186/s12862-015-0442-x (PMC4528382; doi:10.1186/s12862-015-0442-x)

Fig. S2. Figures showing weak relationships between pairwise F_{ST} and geographic distance (a), and pairwise F_{ST} and divergence in colour morph composition (b).

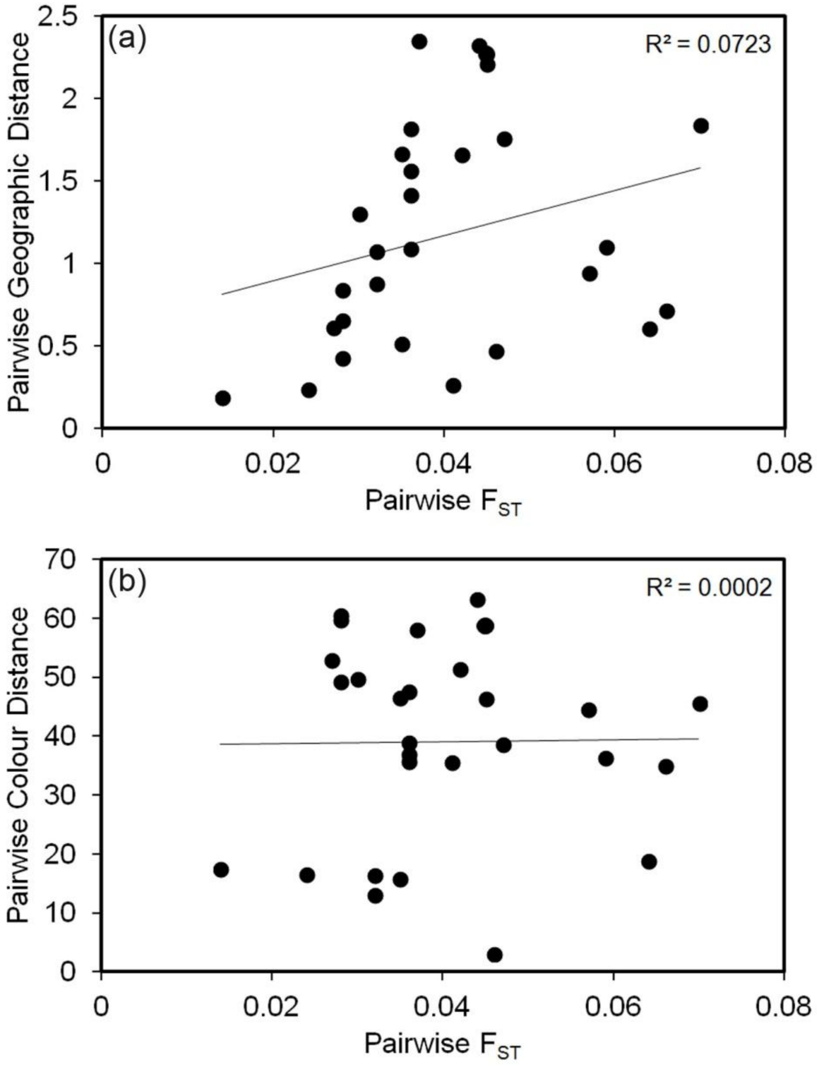

Supplement: Additional file 7: Figure S2. — Figures showing weak relationships between pairwise FST and geographic distance (a), and pairwise FST and divergence in colour morph composition (b). (PDF 152 kb) [file 12862_2015_442_MOESM7_ESM.pdf]
