# Supplementary material for: Environment, but not genetic divergence, influences geographic variation in colour morph frequencies in a lizard
Source: BMC Evol Biol. 2015 Aug 8;15:156. doi: 10.1186/s12862-015-0442-x (PMC4528382; doi:10.1186/s12862-015-0442-x)
Supplement: Additional file 8: Table S6. — Results of four separate multiple regression analyses with the frequency of each colour morph (orange, orange-yellow, yellow and grey) as the dependent variables and mean annual aridity index (aridity) and proportion cover of vegetation <1 m in height (vegetation) as the predictor variables. Statistically significant relationships after false discovery rate correction for multiple tests are bold and italicised [52]. (PDF 88 kb) [file 12862_2015_442_MOESM8_ESM.pdf]

Table S6. Results of four separate multiple regression analyses with the frequency of each colour morph (orange, orange-yellow, yellow and grey) as the dependent variables and mean annual aridity index (aridity) and proportion cover of vegetation <1m in height (vegetation) as the predictor variables. Statistically significant relationships after false discovery rate correction for multiple tests are bold and italicised (Verhoeven et al., 2005).

Morph	Variable	Coefficient	t Value	P > |t|	Standardised Coefficient*
Orange	aridity	-0.752	-4.964	***0.004***	-0.892
	vegetation	-0.078	-0.696	0.518	-0.125
Orange-Yellow	aridity	-0.143	-0.515	0.629	-0.132
	vegetation	0.662	3.229	0.023	0.830
Yellow	aridity	0.843	3.844	***0.012***	0.858
	vegetation	-0.222	-1.370	0.229	-0.306
Grey	aridity	-0.051	-0.236	0.823	-0.070
	vegetation	-0.403	-2.525	0.053	-0.744

*The standardised coefficients allow assessment of the relative strength (slope) of each variable.
